# Biogenic-Mediated Synthesis of Mesoporous Cu_2_O/CuO Nano-Architectures of Superior Catalytic Reductive towards Nitroaromatics

**DOI:** 10.3390/nano10040781

**Published:** 2020-04-18

**Authors:** Mosaed S. Alhumaimess, Amr A. Essawy, Mahmoud M. Kamel, Ibrahim Hotan Alsohaimi, Hassan M. A. Hassan

**Affiliations:** 1 Chemistry Department, College of Science, Jouf University, P.O. Box 2014, Sakaka 42421, Saudi Arabia; aaessawy@ju.edu.sa (A.A.E.); mmkamel@ju.edu.sa (M.M.K.); hmahmed@ju.edu.sa (H.M.A.H.); 2 Chemistry Department, Faculty of Science, Fayoum University, Fayoum 63514, Egypt; 3 Department of Chemistry, Faculty of Science, Suez University, Suez 43511, Egypt

**Keywords:** biogenic synthesis, pomegranate seeds extract, Cu_2_O/CuO nanoparticles, aniline, nitrobenzene reduction

## Abstract

Cu_2_O/CuO nano-architectures were prepared by biogenic-mediated synthesis using pomegranate seeds extract as the reducing/stabilizing mediator during an aqueous solution combustion process of the Cu^2+^ precursor. The fabricated Cu_2_O/CuO nanocomposite were characterized by X-ray diffraction (XRD), Fourier transform infrared spectroscopy (FTIR), X-ray photoelectron spectroscopy (XPS), field emission scanning electron microscopy (FESEM) and nitrogen sorption. Nitrobenzene (NB) was applied a probe to test the catalytic activities of the fabricated Cu_2_O/CuO nanocomposite. The results indicated that pomegranate seeds extract (PSE) manifest Cu_2_O/CuO NPs of tiny particle size, larger pore volume and greater surface area compared to the bulky CuO synthesized in the absence of PSE. The surface area and total pore volume of Cu_2_O/CuO NPs were 20.1 m^2^ g^−1^ and 0.0362 cm^3^ g^−1^, respectively. The FESEM image shows the formation of broccoli-like architecture. The fabricated Cu_2_O/CuO nanocomposite possesses surprising activity towards the reduction of nitro compounds in the presence of NaBH_4_ into amino compounds with high conversion (94%). The reduction process was performed in water as a green solvent. Over four consecutive cycles the resulting nanocomposite also exhibits outstanding stability. In addition, the resulting Cu_2_O/CuO nanocomposite suggested herein may encourage scientists to start preparing more cost-effective catalysts for marketing instead of complicated catalysts.

## 1. Introduction

Metal oxide nanomaterials [1,2] have acquired much interest in the recent past and have activated numerous synthetic avenues. The diverse range of performance and versatility of such nanomaterials greatly encouraged their development and also facilitated the tailoring of metal oxide nanoparticles to satisfy the appropriate size and morphology. The fabrication of nano-architecture of metal oxides [3], such as cobalt, zinc, tin, copper oxides, etc., is of conspicuous advantage due to their improved catalytic efficiency, non-toxicity, positive economic and upscale fabrication approaches. Due to their diversified implementations such as gas sensing [4], solar cells [5], catalysis [6] and electrode materials [7]. The synthesis of Cu_2_O nanoparticles is of particular importance in the scientific as well as the industrial scope.

The unparalleled features of Cu_2_O nanoparticles (NPs) have been harnessed largely for environmental cleanup. Cu_2_O is a *p*-type semiconductor with a humble band gap of about 2 eV, rendering it an essential component in countless applications. Conventionally Cu_2_O nanoparticles are fabricated either through pure copper oxidation (thermal) or through Cu^2+^ ions reduction. There are a number of reports in literature applying various approaches for the synthesizing of Cu_2_O nanoparticles including electrochemical deposition [8], microwave irradiation [9], hydrothermal technique [10], liquid phase fabrication [11,12,13,14,15], seed-mediated method [16] and microemulsions [17].

These were all exploited to prepare Cu_2_O NPs of distinguishable morphologies and sizes, as well as to regulate these variables in certain strategies. From these approaches, it is important to emphasize liquid-phase preparation, since they are easy to perform, do not need high-cost laboratory equipment or deal with hazardous materials, are cheap and therefore less time consuming. Most synthesis in this approach usually begins by adding NaOH to copper (II) precursors [18], and then adding a reducing agent to acquire Cu_2_O nanoparticles. A surface active agent may also be introduced to regulate the size of the particles [19,20]. The predominant drawbacks of the chemical approach for the preparation of Cu_2_O NPs are the use of drastic and costly chemical materials that harm the environment and are not biocompatible. Nanoclass of Cu_2_O nanoparticles further enriches the inquisitiveness of the scientific community and inspires them to establish novel routes for their preparation along with minimal use of cruel chemicals to protect the environment. Scientific development is therefore directly to the environment, a replicable, simple, ecofriendly biological fabrication of sustainable and economical nanoparticles has recently considered a rise in the architecture of green approaches to synthesize a broad spectrum of nanoparticles using both biological extracts and wastes. Extracts stemming from plant parts were also utilized to fabricated nanoparticles as they serve as stabilizing/capping agents and reducing agents respectively. Both biological extracts contain components, like polyphenols, flavones and carbohydrates, which act as a reducing agent or a stabilizer.

Due to their flexibility in various organic reactions, preparation of aniline compounds is of great significance in the industrial processes [21]. The most effective and hands-on approach for aniline synthesis is nitro-compound hydrogenation. While hydrogenation of certain nitro compounds is accomplished facially with different industrial catalysts, it is challenging to selectively reduce the nitro moiety with hydrogen in the presence of other reducible groups. It is therefore highly attractive to develop a reliable catalytic system for the selective reduction of nitro compounds. In the previous decades, material scientists have provided considerable importance to substances comprising copper as a significant organic reaction catalyst [22,23].

Herein, the pomegranate seed extract is provided as the capping reducing/stabilizer for the fabrication of Cu_2_O/CuO nano-architectures during a one pot aqueous solution combustion process of the Cu^2+^ precursor. The synthesized materials were used for the reduction of nitro aryl compounds in the presence of NaBH_4_ to the corresponding amino compounds.

## 2. Experiments 

### 2.1. Materials 

Copper nitrate trihydrate (Cu(NO_3_)_2_·3H_2_O), nitrobenzene (NB), 4-bromo nitrobenzene, 4-nitrobenzaldyde, 2-(2-methyl-5-nitro-1H-imidazol-1-yl) ethan-1-ol, metrnidazole (MET), 1- chloro-3-(2-methyl-5-nitro-1H-imidazol-1-yl) propan-2ol, ornidazole (ORN) and aniline were obtained from Sigma Aldrich (St. Louis, MO, USA). Sodium borohydride (NaBH_4_) was obtained from Merck (Darmstadt, Germany). All experiments were performed using Deionized water.

### 2.2. Methods

#### 2.2.1. Preparative Route for Pomegranate Seeds Extract (PSE)

In 1.0 L pyrix beaker, 150 g of pomegranate seeds collected from fresh fruits were soaked in 400 mL ethanol and kept in refrigerator for 4 days. The ethanol color turns into deep red as a result of extracting the colored biogenic seeds ingredient. After that, this solution was filtered where the resultant clear solution was subjected to rotary evaporation at a working temperature 50 °C. After ethanol evaporation, highly viscous PSE was obtained and stored in ambient temperature until its usage in the proposed biogenic synthetic route.

#### 2.2.2. Bulk Synthesis of CuO (Bulk Fabricated CuO)

Bulk fabricated CuO as a control catalyst was prepared using a traditional combustion approach. A certain amount of copper nitrate (Cu(NO_3_)_2_·3H_2_O) was put in a crucible followed by calcination in static air at 300 °C for 2 h [24].

#### 2.2.3. Biogenic Synthesis of Copper Oxide Nanoparticles (PSE-Capped Cu_2_O/CuO)

In one pot reaction mixture, 100 mg of pomegranate seeds extract (PSE) was put in a crucible (50 mL capacity), and then precisely 30.0 mL of DI water was introduced to solubilize the extract. A certain amount of Cu(NO_3_)_2_·3H_2_O (1.0 g) were added to the crucible upon complete dissolution of extract, agitated until complete dissolution. Through introducing the copper nitrate suggesting an attribute of interaction between PSE and Cu^2+^ ions, noticeable darkness of the pink color of PSE solution was observed. The crucible was then put in a preheated muffle furnace at 300 °C, starting a two-hour solution combustion reaction. At the completion of combustion process Cu_2_O/CuO NPs was produced. The final product was collected and stored in a desiccator without any further purification being done.

### 2.3. Reduction of Nitrobenzene

The reduction of nitrobenzene (NB) using NaBH_4_ was chosen as a model test reaction to assess the catalytic efficiency in the liquid phase of both bulk fabricated CuO and biogenic-mediated synthesis Cu_2_O (PSE-capped Cu_2_O/CuO). In the model experiment, the plastic tube contained 5 mL of 2.5 × 10^−4^ mol L^−1^ NB, 5 mL of deionized water, and 5 mg of NaBH_4_ dispersed in 1 mL ice water were mixed. To that mixture, 1 mg catalyst has been added to this solution. Under ambient conditions, the development of the reaction was monitored by analyzing a small amount of the mixture at a certain time interval. The NB concentration was measured in the Ultraviolet and visible (UV vis-spectrophotometer) Agilent Cary 60 (Agilent Technologies, Stevens Creek Blvd, Santa Clara, USA).

To investigate the durability, the catalyst was removed from the reaction mixture by centrifugation and washed several times with distilled water–ethanol to remove of any adsorbed product. The catalyst was then added to another freshly made NB and NaBH_4_ mixture to begin the next reaction cycle. The reusability experiment was performed for four catalytic rounds.

### 2.4. Characterization

The FTIR findings were reported using a Shimadzu IR Tracer-100 Fourier Transform Infrared Spectrophotometer (Shimadzu, 1 Nishinokyo Kuwabara-cho, Nakagyo-ku, Kyoto 604-8511, Japan). The powder XRD findings were analyzed with an X-ray wavelength Cu-K*α* radiation using the X-ray diffractometer Maxima–X (D/Max2500VB2+/Pc, Shimadzu Company, Kyoto 604-8511, Japan). X-ray photoelectron spectroscopy (XPS) analysis on K-ALPHA (Thermo Fisher Scientific GmbH, Dreieich, Germany) was performed. The morphological characteristics of the fabricated materials were explored using field emission scanning electron microscopy (Zeiss FESEM Ultra 60, 5 kV, Carl Zeiss Strasse 2273447 Oberkochen, and Germany). The material surfaces were coated with Au thin layer for 30 s at 30 mA in sputter coater. The surface textural features comprising surface Area, pore volume and pore width were realized by analyzing the nitrogen adsorption–desorption isotherms at 77 K using A NOVA 4200e (Quantachrome Instruments, Quantachrome GmbH & Co. KG, Boynton Beach, USA). Using the Agilent Cary 60 spectrophotometer, UV-visible spectra were collected. 

## 3. Results and Discussion

### 3.1. X-Ray Diffraction (XRD)

**Figure 1:**
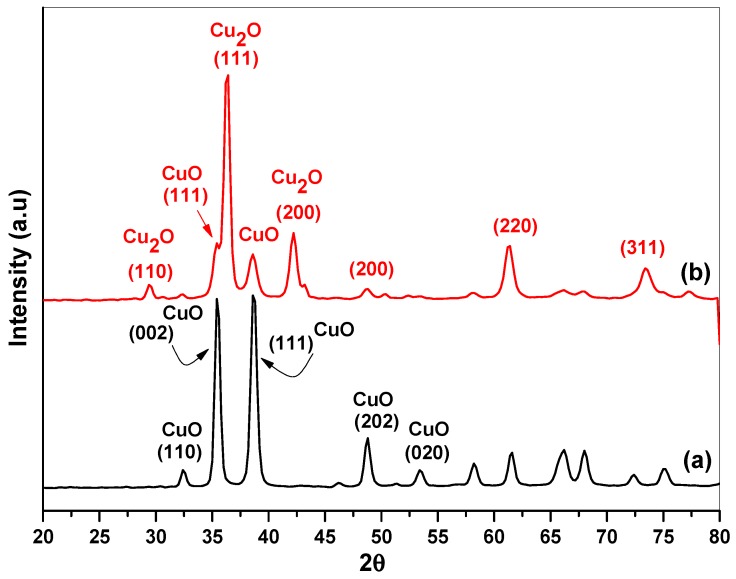
X-ray diffraction patterns of (**a**) bulk fabricated CuO and (**b**) pomegranate seeds extract (PSE)-capped Cu_2_O/CuO materials fabricated by the aqueous solution combustion approach.

The XRD technique was implemented to explore the disparity between copper oxide in crystal and chemical structures. Figure 1 displays the XRD patterns of bulk fabricated CuO compared with biogenic-mediated synthesis Cu_2_O (PSE-capped Cu_2_O/CuO). For bulk fabricated CuO, the XRD pattern revealed the distinctive peaks at 32.4°, 35.5°, 38.8°, 48.7°, 53.3°, 58.3°, 61.5°, 66.2°, 68.10°, 72.4° and 75.2° pertaining for (100), (002), (101), (102), (110), (103), (200), (112), (201), (004) and (202) faces, respectively. The noted diffractions are consistent with Joint Committee on Powder Diffraction Standards (JCPDS No. 45-0937) and correlated with a generic monoclinic structure of CuO. For the PSE-capped Cu_2_O/CuO, the pattern fit the cubic structure of Cu_2_O representing (110), (111), (200), (311) and (222) faces (JCPDS No. 05-0667). In addition, two distinct CuO diffraction peaks with (002) and (111) faces were also manifested and it could be attributed to the ineluctable oxidation during the synthesis or drying steps. Additionally, no diffraction peaks correlated with impurities were observed. 

Due to the competent capping of pomegranate seed extract (PSE) to regulate the size, a substantial broadening of PSE-capped Cu_2_O/CuO than that bulk CuO could be detected. This approximately bigger amorphous feature of PSE-capped Cu_2_O/CuO contributes to its superiority as a catalyst for incalculable applications.

By applying the Debye–Scherrer Equation (1), the mean crystallite size was evaluated:

*D* = *Kλ*/*β cosθ* (1)

where *D* is the crystallite size (nm), *k* is the Scherrer’s constant of value 0.89, *λ* is the X-ray wavelength (*λ* = 1.54056 Å), *β* is the full width of the assessment peak at half maximum (in radian) and *θ* is Bragg’s angle. Consequently, the mean crystallite size for bulk CuO and PSE-capped Cu_2_O/CuO were 17.5 and 10.1 nm respectively. 

### 3.2. FTIR Spectroscopy

**Figure 2:**
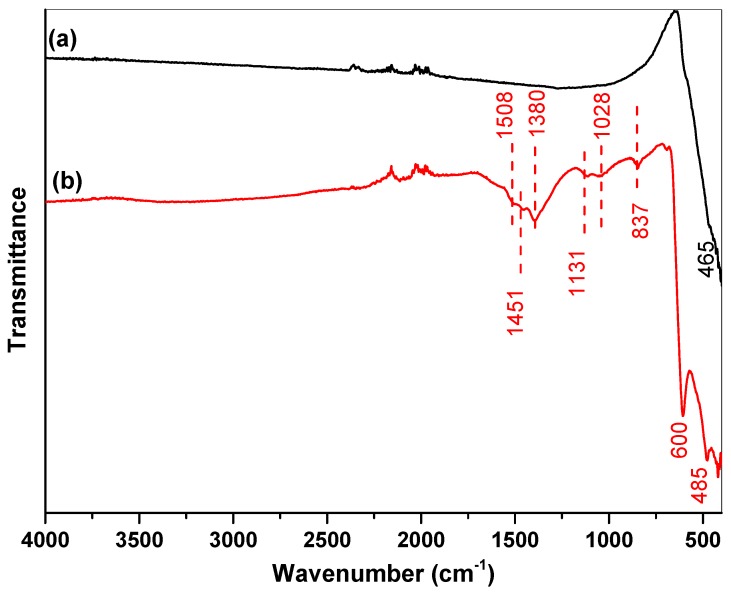
FT-IR spectra of (**a**) bulk fabricated CuO and (**b**) PSE-capped Cu_2_O/CuO materials fabricated by the aqueous solution combustion approach.

The FTIR spectra of bulk fabricated CuO and PSE-capped Cu_2_O/CuO are depicted in Figure 2. The scale ranged between 400 and 4000 cm^−1^, the metal oxide peaks were usually less than 1000 cm^−1^ [25]. In the FTIR spectrum of bulk fabricated CuO (Figure 2a), the weak peak obtained at 465 cm^−1^ is assigned to Cu–O stretching vibration [25]. Furthermore, no further peaks in the range of 605 to 660 cm^−1^ were detected, which completely excluded the presence of another phase such as Cu_2_O [26,27]. In the FTIR spectrum of PSE-capped Cu_2_O/CuO (Figure 2b), band at 600 cm^−1^ was related to Cu(I) –O stretching vibration, while the peak at 485 cm^−1^ was attributed to Cu(II) –O stretching vibration. Accordingly, the FTIR findings supported the fabrication of Cu_2_O along with CuO for the sample prepared by biogenic-mediated approach. The vibrations in the region 1500–800 cm^−1^ could be attributed to the observation of organic residues from the biogenic-mediated.

### 3.3. X-Ray Photoelectron Spectroscopy (XPS)

**Figure 3:**
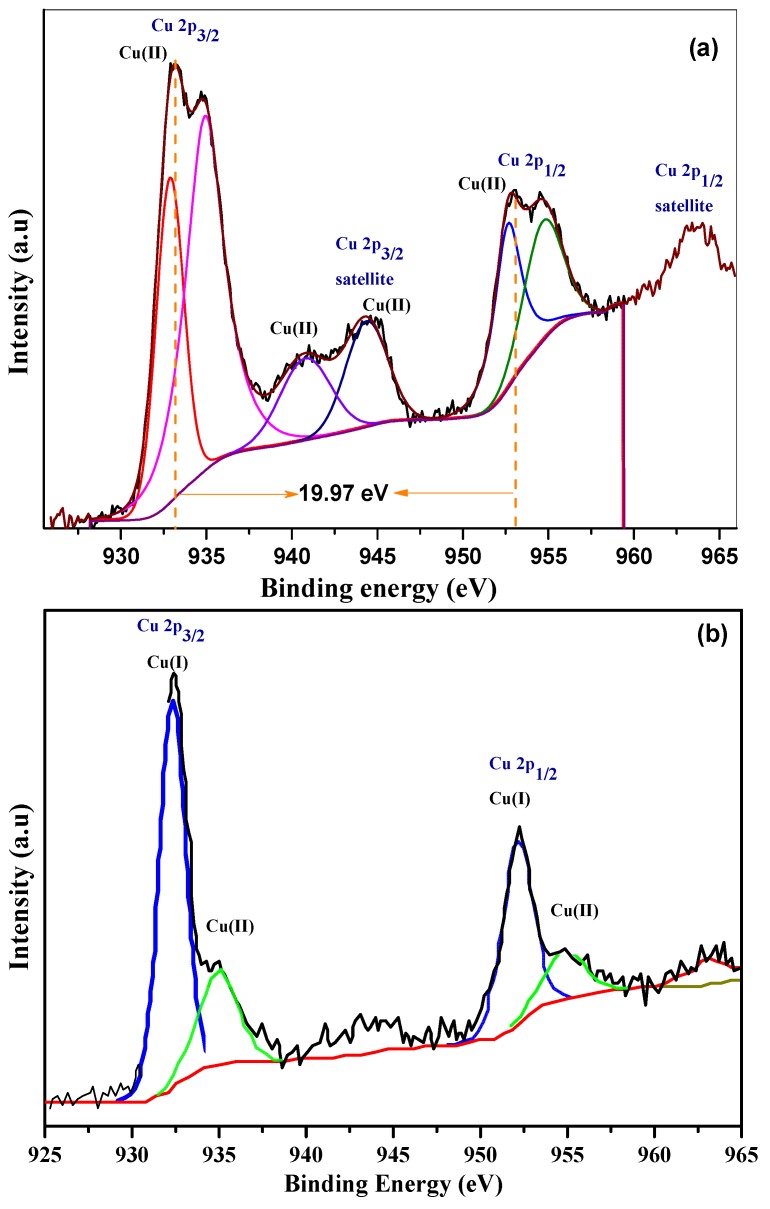
Deconvoluted XPS spectra of Cu 2p of (**a**) bulk fabricated CuO and (**b**) PSE-capped Cu_2_O/CuO materials fabricated by the aqueous solution combustion approach.

X-ray photoelectron spectroscopy (XPS) was investigated owing to the preciseness inspection of the surface oxidation state of the fabricated materials. Figure 3 manifests the high resolution XPS spectra of the bulk fabricated CuO and PSE-capped Cu_2_O/CuO. Figure 3a shows the nominated Cu 2p in bulk fabricated CuO manifesting two discrete peaks at binding energies of 934.1 and 952.9 eV, assigning to Cu 2p_3/2_ and Cu 2p_1/2_ respectively [28]. The length between such two Cu 2p peak values was 19.97 eV, which is quite similar to previous CuO spectrum studies [29]. In addition, with the wide satellite peaks at greater binding energy than the principal peaks, CuO was further confirmed. The predominant peak of Cu 2p_3/2_ at 934.1 eV was followed by two satellite peaks on the greater binding energy at around 941.03 and 944.50 eV, suggesting the existence of CuO [29]. Approximately 9.6 eV disunited Cu 2p_1/2_ predominant peak at 952.9 eV and its satellite at 962.5 eV that also evinced the formation of CuO nanoparticles. Due to numerous plant metabolites such as carbohydrates, phenolic and flavonoid, the use of plant extract for fabrication performed an important task in metal ion bioreduction, producing nanoparticles. The plant serves as bioreactors, thus affecting the fabrication of nanoparticles through metal ions reduction [30,31]. Figure 3b shows the detailed XPS of Cu 2p in biogenic-mediated synthesis Cu_2_O/CuO manifesting two recognizable peaks at binding energies of 932.5 and 952.8 eV, referring to the main Cu 2p_3/2_ and Cu 2p_1/2_, respectively. These core levels could be resolved into two, Cu (I) as a major and Cu(II) as minor species. In the Cu 2p spectrum, no satellite peaks were detected to somehow validate the purity of Cu_2_O/CuO. Having regard to the XPS results demonstrating the coexistence of Cu^1+^ as a major species and Cu^2+^ as a minor species on the material surface, one is willing to concede that such combined ionic species of various oxidation states that appear on the surface of the materials may create a great environment for enhancing catalytic efficiency, facilitating the redox process. Moreover, the analysis of XRD and XPS precisely indicates the chemical integrity of Cu_2_O fabrication. 

### 3.4. Morphological Features Using FESEM

**Figure 4:**
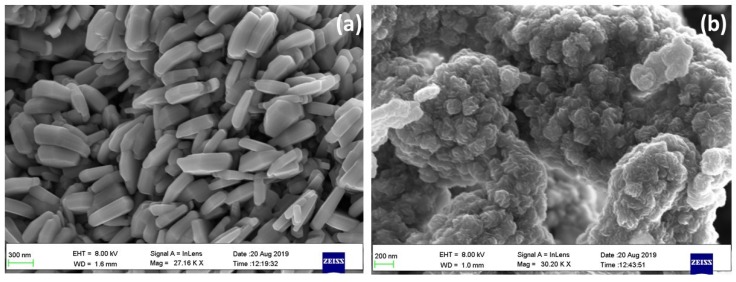
FESEM images of (**a**) bulk fabricated CuO and (**b**) PSE-capped Cu_2_O/CuO materials fabricated by the aqueous solution combustion approach.

Using FESEM imaging as illustrated in Figure 4, more morphological inspection of the bulk fabricated CuO and PSE-capped Cu_2_O/CuO was presented. The typical image for bulk fabricated CuO is demonstrated in Figure 4a while the FESEM image of PSE-capped Cu_2_O/CuO sample depicts mainly broccoli like morphology with high porosity as shown in Figure 4b. It is obvious from FESEM findings that the developed aqueous solution combustion technique demonstrated a fascinating surface morphology in the existence of the biogenic mediated reduction and capping PSE reagent. 

### 3.5. Surface Characteristics

**Figure 5:**
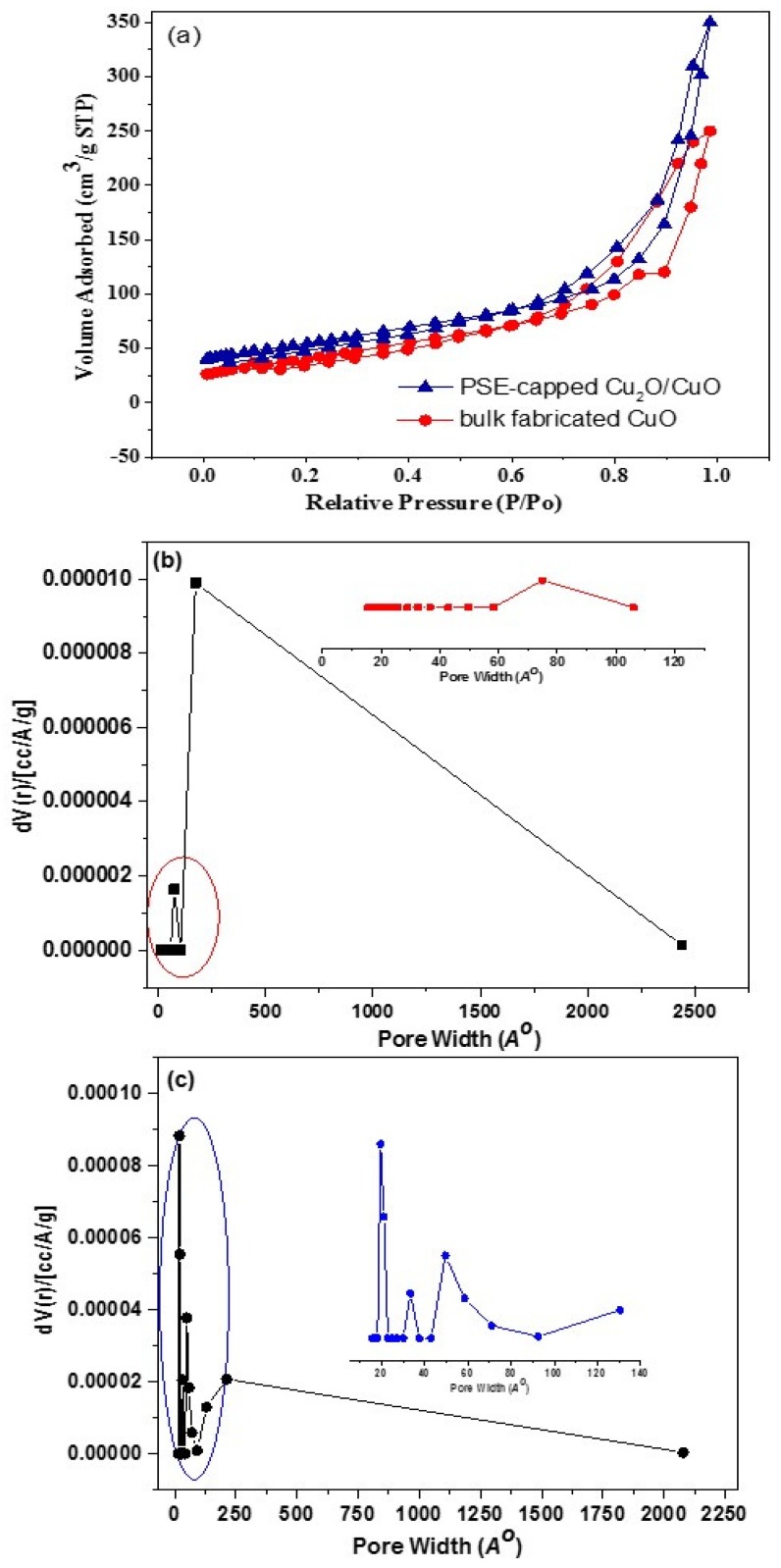
(**a**) N_2_ adsorption/desorption isotherms and pore size distribution of (**b**) bulk fabricated CuO and (**c**) PSE-capped Cu_2_O/CuO materials fabricated by the aqueous solution combustion approach.

Since the catalytic processes occur in the inner solid surface, situated within the pores, modulation of pore size is critical for the transfer of mass and the diffusion of reactants to active sites. Not only are pores the routes for reactants and products but they also impact the incorporation of reactive metals during catalyst fabrication. For small pores (micropores), the catalytic activity is restricted to diffusion while less active surface area is accessible for broader pores (macropores). Therefore, surface characteristics such as surface area, pore volume and pore width must follow the requirements needed for a longer life of catalysts. To tackle the importance of PSE in determining the surface features of the fabricated materials, N_2_ adsorption–desorption isotherms were performed and presented in Figure 5a. The isotherm profiles of the fabricated materials belong to type II isotherms with H3 hysteresis loops, according to IUPAC classification. The isotherms are related mainly to the existence of particles with slit-shape pores. Table 1 lists the surface area, pore volume and pore width values for PSE-capped Cu_2_O/CuO fabricated via biogenic approach compared to bulk fabricated CuO as a control material that synthesized without PSE. It is worth noting, the introduction of pomegranate seed extract could result in a 2-fold increase in the surface area and pore volume. In addition, Figure 5b,c displayed the pore size distribution of bulk fabricated CuO and PSE-capped Cu_2_O/CuO. As illustrated, a mesoporous structure was noticed in the PSE-capped Cu_2_O/CuO sample developed using a biogenic approach. The unique surface and textural structure provided by the use of pomegranate seed extract is therefore designed to save a wide range of possible space active sites.

### 3.6. Catalytic Properties

#### 3.6.1. Influence of Reaction Time

**Figure 6:**
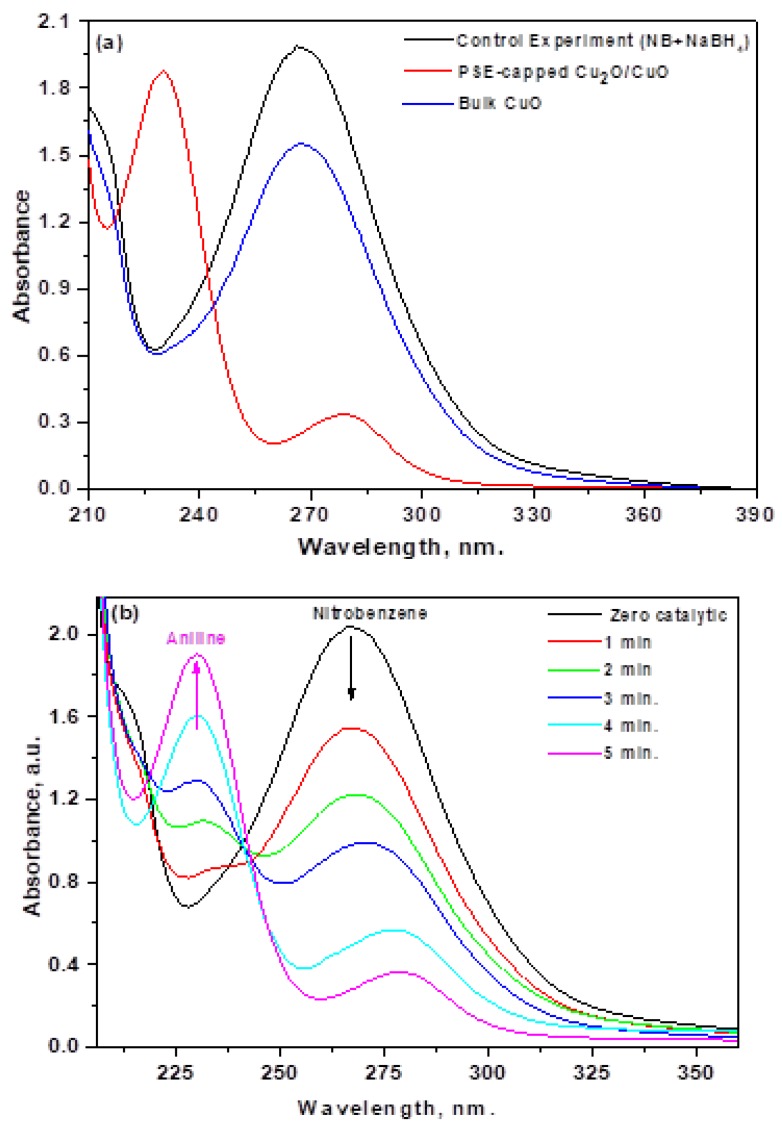
Catalytic properties of the synthesized catalysts. (**a**) UV-vis absorption spectra of the reduction of NB over bulk fabricated CuO and PSE-capped Cu_2_O/CuO catalysts and (**b**) UV-vis absorption spectra displaying gradual reduction of NB over PSE-capped Cu_2_O/CuO (1 mg catalyst, 5 mg NaBH_4_, 2.5 × 10^−4^ mol L^−1^ NB).

It is predominantly recognized that catalytic performance is improved by raising the surface area-to-volume proportion of nanoparticles or through enhancing the availability of the metal surface to the reactants molecule by tuning the surface oxidation state of the materials fabricated [32,33]. While the traditional approaches for the production of bulk materials have been shown to lessen the potency of these particles for catalytic purposes, we intentionally obstructed this in our approach. Rather, we purpose to use the biogenic-mediated synthesis through interacting pomegranate seed extract with the surface of nanoparticles. Additionally, the nanoparticles produced were really small with multiple oxidation state, growing their aptitude as a nanocatalyst. The catalytic performance of those fabricated nanoparticles was evaluated applying the model to reduce nitrobenzene (NB) to aniline in the existence of sufficient sodium borohydride [34,35,36]. As manifested in Figure 6, the time-based UV-vis absorption spectroscopy of the liquid phase reduction of NB might be readily tracked. As a control experiment in the absence of the catalyst, the mixture of NB and NaBH_4_ displayed a strong peak at 270 nm owing to the absorption of NB, demonstrating that the reaction did not occur in the absence of the catalyst. After the catalysts were introduced into the reaction mixture, the NB absorption peak at 270 nm decreased significantly with a complementary peak increase at the maxima 230 nm (high-energy) and at 280 nm (low-energy), due to the formation of aniline product. A comparatively feeble decline in the absorption spectrum of NB corresponding to 20.9% was achieved when using bulk-fabricated CuO as a catalyst. This is owing to a great affinity for the adsorption of NB on CuO surface through the strong interaction between a highly positive charge Cu(II) ions on the surface and electron environment of NO_2_-groups of NB. On the contrary, for PSE-capped Cu_2_O/CuO, the absorption spectrum of NB mostly disappeared with a reduction efficiency of 94% (Figure 6a). It should be observed that the PSE-capped Cu_2_O/CuO displayed the greatest catalytic efficiency to complete the reduction of NB with only 5 mins (Figure 6b). This could be attributed to the combination between Cu(I) and Cu(II) species significantly affecting the reduction performance. This finding also emphasizes that the actual active species in the reduction of NB was Cu(II)/Cu(I) ion pairs.

#### 3.6.2. Kinetics Studies

**Figure 7:**
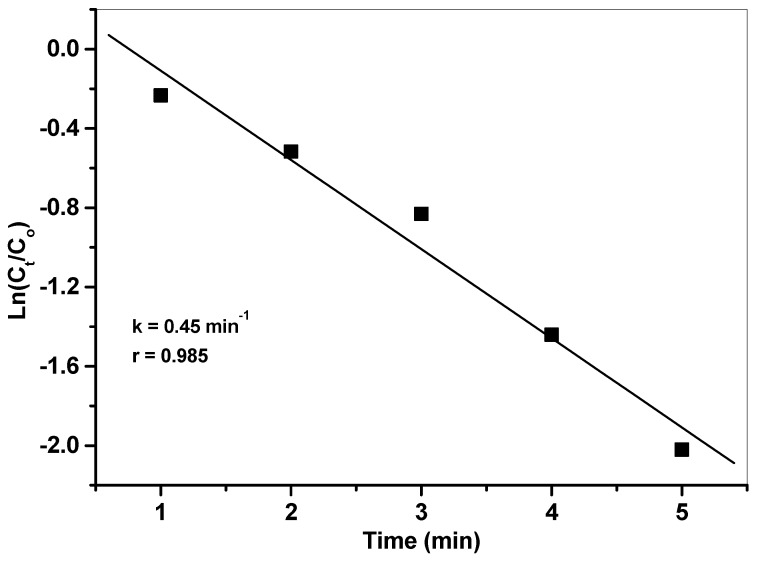
Kinetics plot of ln(*C_t_*/*C*_0_) of NB reduction versus time using a PSE-capped Cu_2_O/CuO catalyst.

The catalytic reduction of NB into its subsequent amine can be assessed as a pseudo-first order reaction owing to the high original concentration of NaBH_4_. Consequently, the plot of ln (*C_t_*/*C*_0_) explored the kinetics of the reduction process against the time where *C_t_* and *C*_0_ demonstrated the NB concentrations at any time, *t* after catalyst is added and 0, respectively (Figure 7). For PSE-capped Cu_2_O/CuO, the rate constant (*k*) estimated from the slope of the fitted plot was realized to be 0.45 min^−1^.

#### 3.6.3. Influence of Various Substrates

**Figure 8:**
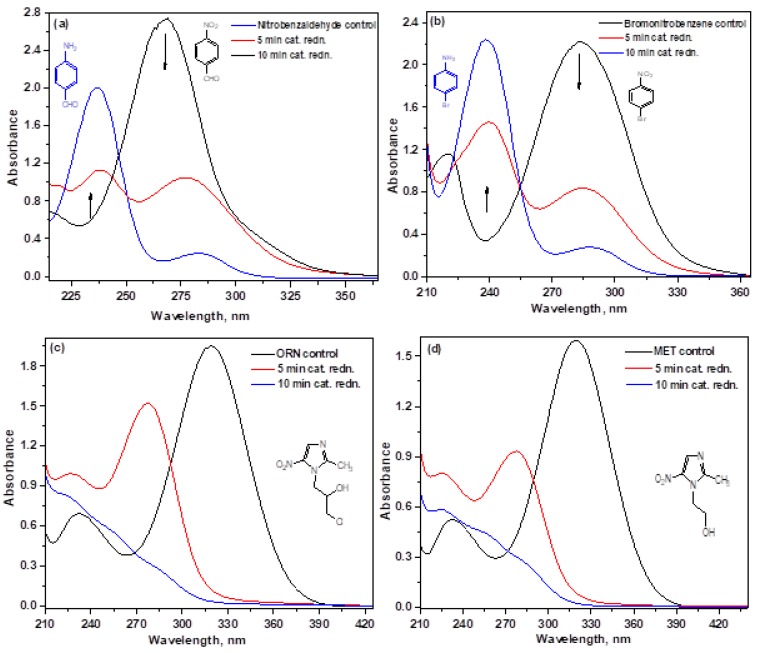
UV-vis absorption spectra of the reduction of various nitro compounds: (**a**) nitrobenzaldehyde, (**b**) 4-bromo nitrobenzene, (**c**) ornidazole and (**d**) metrnidazole over a PSE-capped Cu_2_O/CuO catalyst.

Under the same optimal conditions, various nitro aryl compounds with distinguished electronic identity such as 4-bromo nitrobenzene, 4-nitrobenzaldyde, 2-(2-methyl-5-nitro-1H-imidazol-1-yl) ethan-1-ol and 1- chloro-3-(2-methyl-5-nitro-1H-imidazol-1-yl) propan-2ol were investigated to highlight the impact of the substrate and thoroughness of the catalytic system. Throughout the reaction other functional groups including bromo, formyl, hydroxyl and chloro remain unchanged. It is worth noting that outstanding conversions to the appropriate reduced product were observed for all nitro compounds applied (Figure 8). Therefore, these findings revealed that the fabricated PSE-capped Cu_2_O/CuO was effective and an appropriate catalyst for the reduction of various nitro aromatic compounds with great yield in water. 

#### 3.6.4. Reaction Mechanism

**Scheme 1:**
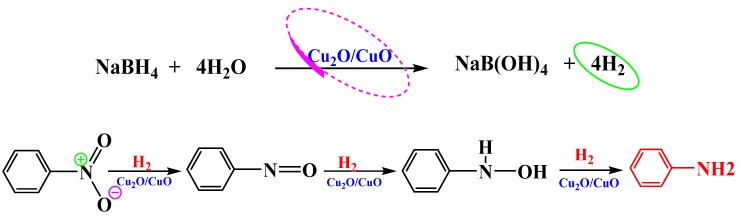
A presumable mechanism on the reduction of the nitro compound to amino compounds catalyzed by the PSE-capped Cu_2_O/CuO catalyst.

Based on the current findings, a probable reaction mechanism (Langmuir–Hinshelwood (L–H)) [37,38] for the nitro compound reduction with NaBH_4_ catalyzed by PSE-capped Cu_2_O/CuO (Scheme 1) was suggested. According to this mechanism the nanostructure materials offered the surface needed for the catalytic reduction process. Five stages in the nitro compounds reduction reactions were manifested including (i) release of the hydrogen by the interaction of a nanocatalyst with NaBH_4_ through the adsorption process, (ii) hydrogen adsorption, (iii) the adsorption of nitro compounds on the surfaces of catalyst, (iv) the transfer of electrons from BH_4_^−^ to nitro compounds and (v) the produced amino compounds were detached from the catalyst surface to make the surface free for the next catalytic cycle to start. The reduction was likely occurring on the active sites of PSE-capped Cu_2_O/CuO nanoparticles through the released hydrogen produced by sodium borohydride decomposition.

#### 3.6.5. Catalyst Recycling

**Figure 9:**
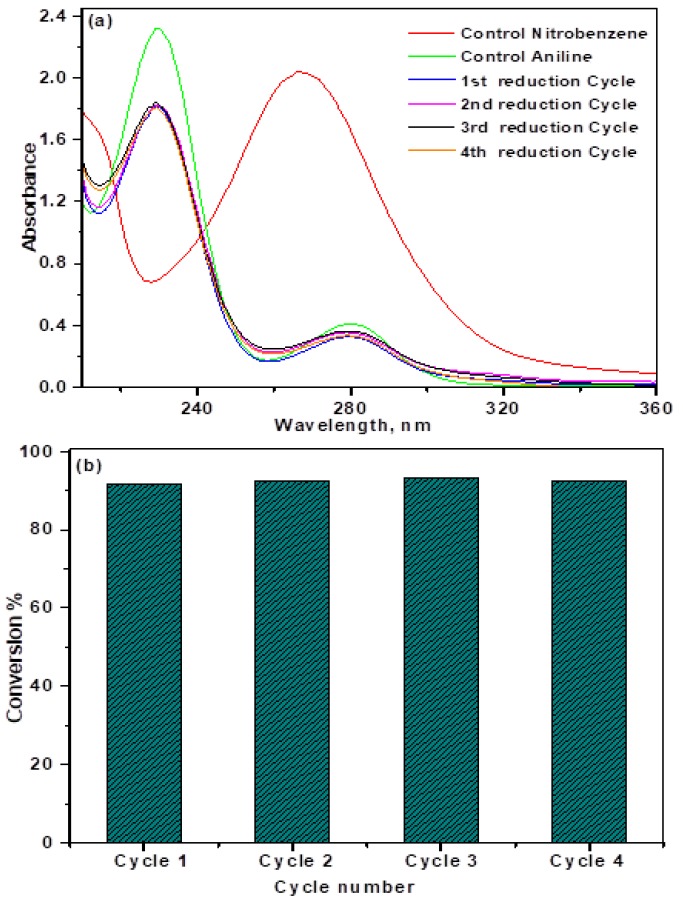
Reusability of the PSE-capped Cu_2_O/CuO catalyst: (**a**) UV-vis absorption spectra of the reduction of NB for successive 4 cycles and (**b**) conversion (%) of NB reduction for each cycle using the PSE-capped Cu_2_O/CuO catalyst.

Due to the exceptional catalytic performance, we inevitably sought to explore the durability of the catalytic system after consecutive exposure to this reduction reaction. The durability of PSE-capped Cu_2_O/CuO as a catalyst for NB reduction was investigated by examining the conversion performance for four consecutive runs as shown in Figure 9. It is worth noting that the catalytic performance was observed four times in the reduction of NB to aniline without an apparent significant loss of their activity. It was confirmed that activity retained was nearly identical after four times of catalyst recycling (first cycle 91.7%, second cycle 92.6%, third cycle 93.1% and forth cycle 92.3% aniline was obtained). The main explanation for the small decrease in activity was catalyst losses during the recovery process.

## 4. Conclusions

It is a very facile approach of the synthesis of PSE-capped Cu_2_O/CuO nanoparticles using pomegranate seeds extract with broccoli like architectures. The current approach offers a very easy and plausibility pathway to fabricate the Cu_2_O/CuO nanocomposite with a surface area of 20.1 m^2^ g^−1^ owing to its great chemical versatility and structural plausibility. The PSE-capped Cu_2_O/CuO nanocatalyst was found to be extremely active and could be recycled without substantial loss of catalytic performance for four sequential cycles.

## Figures and Tables

**Table 1:** Structural parameters for bulk fabricated CuO and PSE-capped Cu_2_O/CuO materials.

Catalysts	*S*_BET_^a^ /m^2^ g^−1^	*V*_BJH_^c^ /cm^3^ g^−1^	*D*_BJH_^b^ /nm
Bulk fabricated CuO	9.2	0.0081	15.2
PSE-capped Cu_2_O/CuO	20.1	0.0362	3.5

^a^ BET surface area, ^b^ Pore volume and ^c^ Pore diameter.
